# A Novel Staging System to Forecast the Cancer-Specific Survival of Patients With Resected Gallbladder Cancer

**DOI:** 10.3389/fonc.2020.01281

**Published:** 2020-07-28

**Authors:** Yongcong Yan, Jianhong Lin, Mengyu Zhang, Haohan Liu, Qianlei Zhou, Ruibin Chen, Kai Wen, Jie Wang, Zhiyu Xiao, Kai Mao

**Affiliations:** ^1^Department of Hepatobiliary Surgery, Sun Yat-Sen Memorial Hospital, Sun Yat-Sen University, Guangzhou, China; ^2^Guangdong Provincial Key Laboratory of Malignant Tumor Epigenetics and Gene Regulation, Sun Yat-Sen Memorial Hospital, Sun Yat-Sen University, Guangzhou, China; ^3^Department of Gastroenterology and Hepatology, The First Affiliated Hospital, Sun Yat-Sen University, Guangzhou, China

**Keywords:** gallbladder cancer, positive lymph node ratio, cancer-specific survival, nomogram, decision curve analysis, surveillance, epidemiology and end results

## Abstract

**Objective:** Gallbladder cancer (GBC) is one of the most aggressive malignant tumors, and there is no effective and convenient method for predicting cancer-specific survival (CSS). We aim to develop a novel nomogram staging system based on the positive lymph node ratio (pLNR) for GBC patients.

**Methods:**A total of 1,356 patients enrolled in the study. We evaluated the prognostic value of the pLNR and built a prognostic nomogram staging system based on the pLNR in the training cohort. The concordance index and calibration plots were used to evaluate model discrimination. The predictive accuracy and clinical value of the nomograms were measured by decision curve analysis (DCA). The CSS nomogram was further validated in an internal validation cohort.

**Results:**The pLNR was an independent prognostic factor for CSS based on Cox regression analyses. A prognostic nomogram that combined T classification, pLNR, M classification, histologic grade, live metastasis, and tumor size was formulated with a c-index of 0.763 (95% CI, 0.728–0.798), while the c-indexes for the staging system of AJCC 8th, 7th, and 6th for CSS prediction were 0.718, 0.718, and 0.717, respectively. The calibration curves showed perfect agreement. The DCA showed that the nomogram provided substantial clinical value. The nomogram (the AUCs for 1, 3, and 5 years were 0.693, 0.716, and 0.726, respectively,) showed high prognostic accuracy.

**Conclusion:**We have developed a formulated nomogram staging system based on the pLNR that allows more accurate individualized predictions of CSS for resected GBC patients than the AJCC staging systems.

## Introduction

Gallbladder cancer (GBC) is an uncommon tumor with an incidence of 2.5 out of 100,000 (1, 2), accounting for ~0.7% of all adult cancers in the USA and 1.2% in China (3, 4). GBC is generally considered rare, but it is the most common malignancy of the biliary tract, accounting for 80–95% of biliary tract cancers (5). GBC is also a highly fatal disease with a median survival of ~6 months, while the 5-year survival rate is only 5% (6–8) because it is often diagnosed late in the process when the tumors are already large enough to cause obstruction and invade nearby structures. For patients with metastasis, the main treatment is chemotherapy-based, and radical cholecystectomy combined with adjuvant therapy is a potential curative treatment for patients with localized disease. Mitin et al. (9) reported that adjuvant chemoradiation improves the survival of GBC-resected patients, except for those diagnosed with stage T1N0 disease. Kasumova et al. (10) proposed that adjuvant chemotherapy with or without radiation provides prolonged survival after the resection of T2/T3 tumors. Thus, an accurate GBC staging system is necessary to guide the subsequent therapeutic strategy for patients who have undergone radical surgery.

The widely used staging system for GBC is the American Joint Committee on Cancer (AJCC) staging system, and lymph node status has been conventionally described as the location of the invaded lymph node or the number of invaded lymph nodes in the AJCC 7th or 8th edition staging system, respectively (11, 12). However, Negi et al. (13) reported that the positive lymph node ratio (pLNR), but not the location or number of invaded nodes, independently predicts the prognosis of patients who have undergone curative resection. For the past few years, pLNR has been proven to be a better predictor of prognosis than positive lymph nodes in various tumors, including colorectal, gastric, pancreatic, esophageal, papillary thyroid, and non-small cell lung cancers (14–19). With respect to GBC, recent studies on the prognosis of pLNR are rare.

We tried to analyze the significance of pLNR in GBC patients and built a novel staging system based on a formulated nomogram for cancer-specific survival (CSS), which was compared with the AJCC 7th and 8th edition staging systems.

## Materials and Methods

### Patients and Study Design

**Figure 1:**
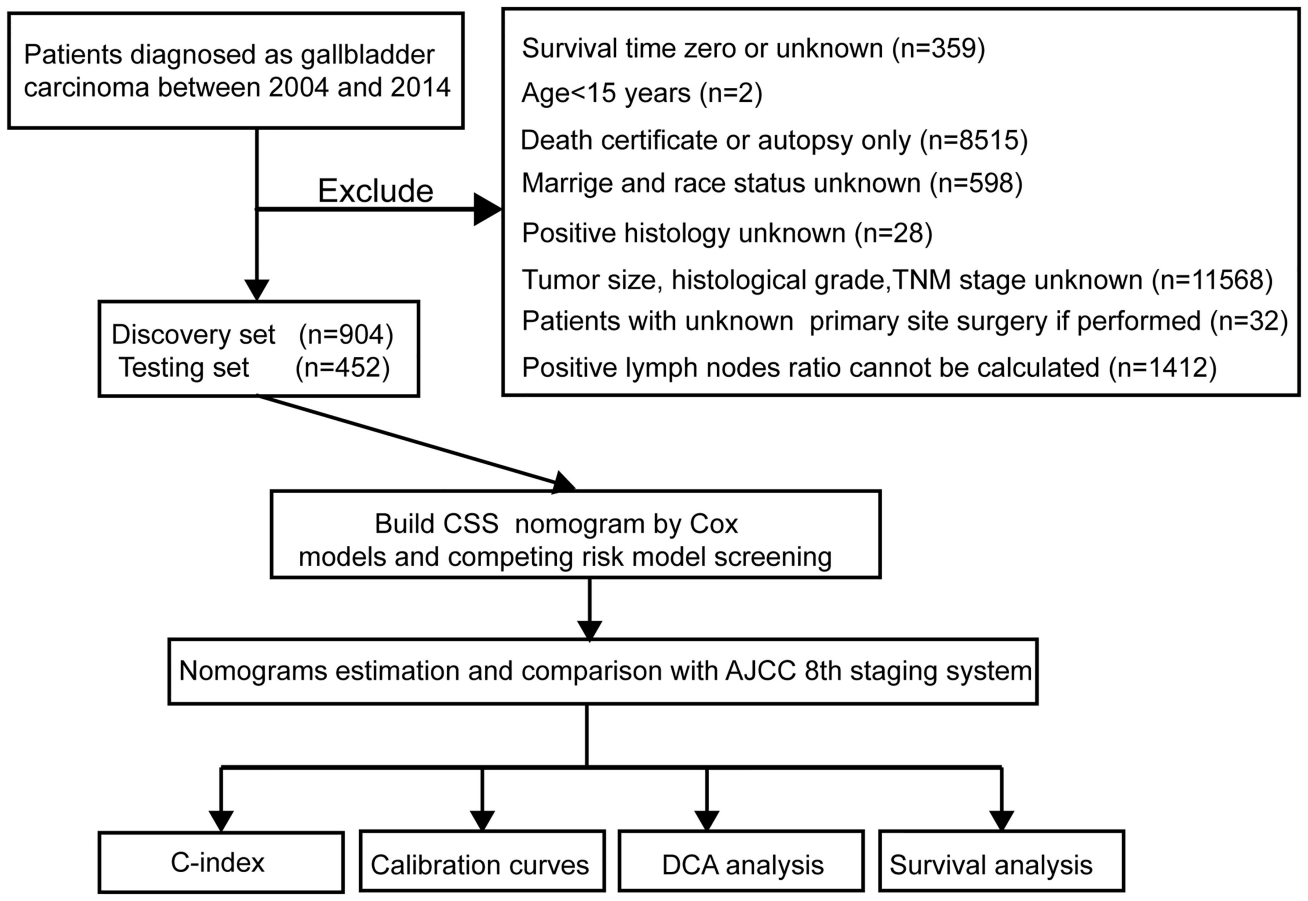
Study flowchart. CSS, cancer-specific survival; DCA, decision curve analysis; TNM, primary tumor, regional lymph nodes and distant metastasis; AJCC, The American Joint Committee on Cancer; C-index, concordance index.

The clinical data related to all patients under the gallbladder heading (Site Recode ICD-O-3/WHO 2008) obtained in this study were rooted mainly in the public SEER database, which is available as open access data. We had permission to access the database with the ID 11889-Nov2017 (permission date: June 5, 2018). The flow chart used for data selection is shown in Figure 1. The following data were received: diagnostic confirmation achieved based on microscopic analysis; patient background characteristics (age, gender, race, and marital status); and tumor-related factors (tumor size and invasion, histologic grade, liver metastasis, lung metastasis, brain metastasis, bone metastasis, regional lymph nodes, positive regional lymph nodes). All patients were randomly divided into a training cohort with N^*^p samples and an internal validation cohort with N^*^(1-p) samples (*p* = 2/3). The pLNR was calculated as the number of positive regional lymph nodes divided by the number of regional lymph nodes examined. This study was approved by the Medicine Institutional Review Board of Sun Yat-sen Memorial Hospital, Sun Yat-sen University.

### Nomogram Construction and Validation

We used a univariate Cox regression analysis to screen for risk clinicopathological factors for CSS in the SEER training cohort. We further performed a multivariate Cox regression analysis to screen for important independent factors for CSS. All variables were screened using the forward stepwise selection method in a Cox multivariate analysis regression model (20, 21). A competing risk model based on risk factors was evaluated by Cox multivariate analysis to ensure that they were related to CSS. A novel nomogram based on the pLNR for predicting CSS at 1 and 3 years was formulated with the other identified independent important factors. The SEER internal validation cohort was used to evaluate the predictive reliability and accuracy of the nomogram. The c-index quantified the discrimination performance between two random patients, with a *c*-index of 0.5 indicating no discrimination and a value of 1 indicating perfect discrimination (22). Calibration plots were generated to validate the accuracy and reliability of the nomogram by comparing the nomogram-predicted and actual survival rates determined in a Kaplan-Meier analysis with 1,000 bootstrap resamples (23).

### Clinical Application Value Assessment

A decision curve analysis (DCA) was performed to identify and compare the clinical application value between the nomogram model and other clinical features by calculating the net benefits at each risk threshold probability (24, 25). The net benefit was determined by subtracting the proportion of all false-positive patients from the proportion of true-positive patients and weighted by the relative harm caused by non-treatment compared with the negative consequences of unnecessary treatment (26). On the basis of the DCAs, we further plotted curves to evaluate the clinical impact of the nomogram to help us more intuitively understand its significance value (27).

### Statistical Analysis

The chi-square test and Student's *t*-test were used to compare categorical variables and continuous variables. The cutoff value of pLNR was determined by the maximum Youden index of the receiver operating characteristic (ROC) curve. Kaplan-Meier survival curves were used to compare CSS among different groups with the survival differences by a two-sided log-rank test in software R version 3.3.4 (www.R-project.org). Univariate and multivariate Cox regressions were performed to generate HRs and 95% CIs in IBM SPSS Statistics version 24 (SPSS, Inc., Chicago, IL, USA). The ROC curve analysis, competing risk, nomogram, *c*-index, calibration plots, DCA, and clinical impact curves were analyzed in software R version 3.3.4 with relevant packages, such as the survivalROC, cmprsk, rms, survival, calibrate, and decision curve packages. The cutoff value of the nomogram model was calculated by X-tile (Yale University, New Haven, CT, USA) (28). All statistical tests were two-sided, and a *P* < 0.05 was considered statistically significant.

## Results

### Study Flowchart and Clinicopathological Characteristics

The study flowchart is shown in Figure 1. A total of 1,356 patients, including a training cohort (*n* = 904) and an internal validation cohort (*n* = 452) diagnosed with GBC, were included and analyzed on the basis of the abovementioned criteria. The baseline clinicopathological features were displayed without significant differences between the two sets (*P* > 0.05, Supplementary Table 1). The median ages (interquartile range) of the patients in the training cohort, the internal validation cohort and the entire SEER cohort were 67.11 (55.16–79.06), 67.26 (55.35–79.17), and 67.16 (55.23–79.09) years, respectively. The median CSS times in the above three sets were also 548 (113–983), 530 (81–979), and 542 (103–981) days, respectively, while the 1-, 2- and 3-year CSS rates were 54.94, 29.65, and 15.12%, respectively, in the entire SEER cohort.

### Prognostic Significance of the pLNR

**Figure 2:**
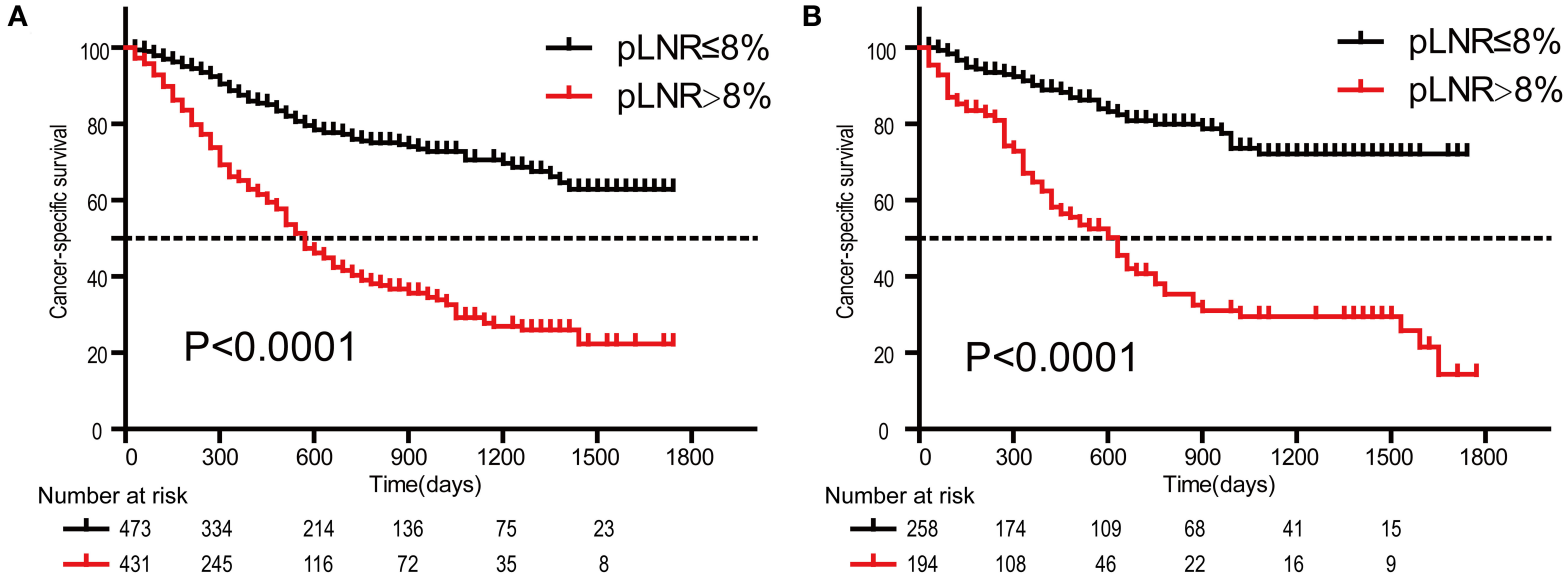
Kaplan-Meier survival curves of cancer-specific survival for patients according to the LNR. The training cohort **(A)**; the validation cohort **(B)**. pLNR, positive lymph node ratio.

**Figure 3:**
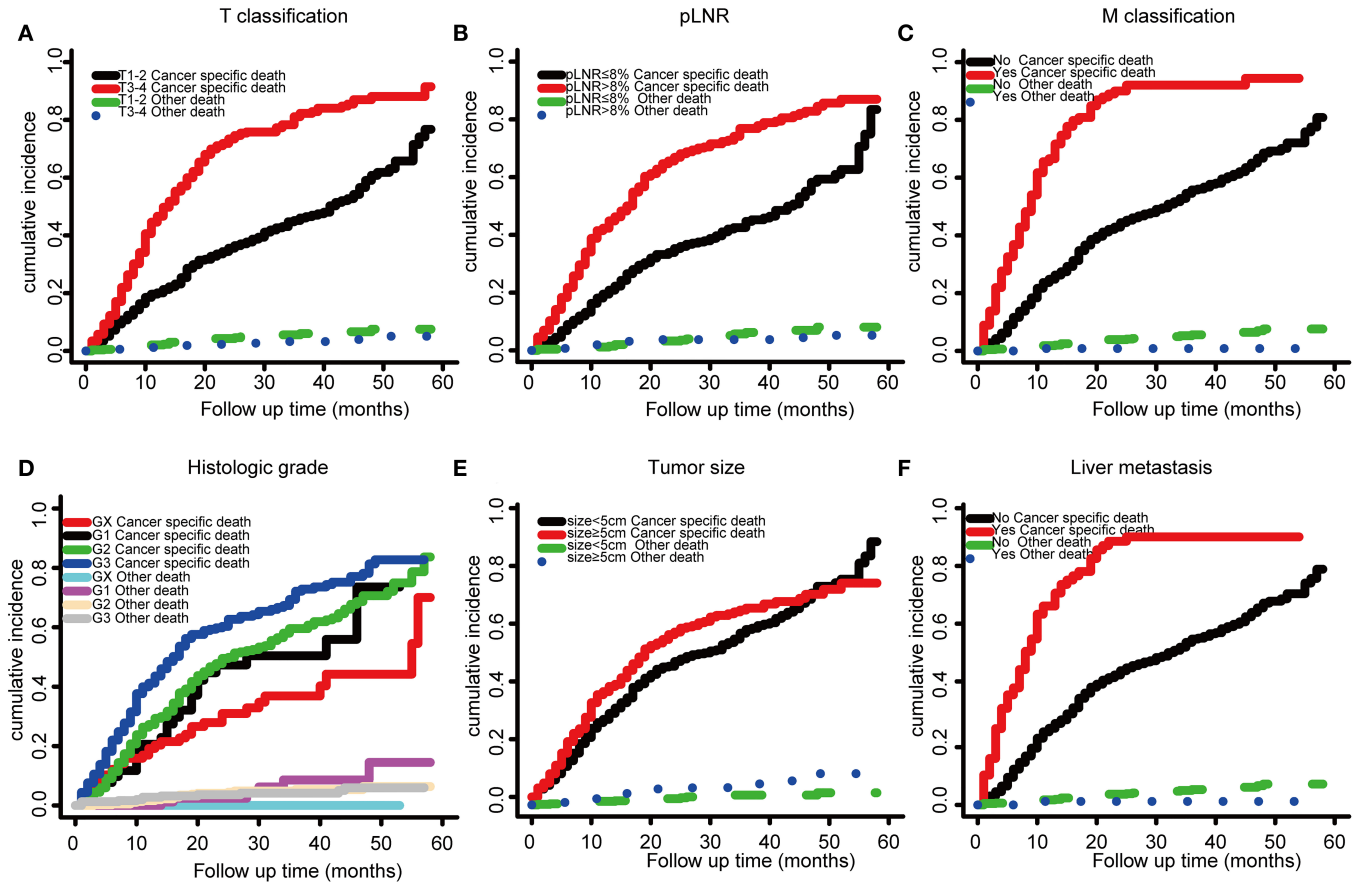
Competing risk model based on risk factors by T classification, **(A)**, LNR **(B)**, M stage **(C)**, histologic grade **(D)**, tumor size **(E)**, and liver metastasis **(F)**.

The optimal cutoff value for pLNR determined by the maximum Youden index of the ROC curve was 0.08. According to the survival analysis, patients in the pLNR>8% group had a poorer CSS than those in the pLNR ≤ 8% group in the training cohort (Figure 2A) and validation cohort (Figure 2B). In the training cohort, the 1-, 2-, and 3-year CSS rates were 62.8, 38.5, and 20.5% in the lower pLNR cohort and 46.9, 22.5, and 9.5% in the higher pLNR cohort, respectively. Similar results were shown in the validation cohort, with 61.2, 35.6, and 18.6% in the lower pLNR cohort and 45.3, 15.9, and 9.7% in the higher pLNR cohort, respectively. pLNR was associated with histologic type, histologic grade, tumor size, tumor extension, N classification, M classification, TNM staging system, and distant organ metastasis (including bone, brain, liver, and lung) by statistical significance in the entire SEER cohort (Supplementary Table 2). As shown in Figure 3, we used a competing risk model to verify the correlation of risk factors (T classification, pLNR, M staging, histologic grade, tumor size, and liver metastasis) with CSS. All six factors were more strongly associated with cancer-specific death than with other causes of death.

### Independent Significant Factors in the Training Cohort

To further identify candidate predictors of CSS, all clinicopathological features were evaluated by Cox proportional hazard regression analysis. As shown by univariate analysis, tumor size, tumor extension, pLNR, liver metastasis, histologic grade, histologic type, TNM staging, T classification, N classification, and M classification were considered significant risk factors in the training cohort (Supplementary Table 3). In the multivariate analysis, eight variables were associated with CSS: histologic grade (G1, HR = 1.33; G2, HR = 1.566; G3, HR = 1.958; *P* = 0.025), AJCC 8th edition stage (II, HR = 1.059; III, HR = 1.944; IV, HR = 4.028; *P* = 0.001), T classification (T3 + T4, HR = 1.888; *P* < 0.001), N classification (N1, HR = 0.211; N2, HR = 0.315; *P* = 0.005), M classification (M1, HR = 1.696; *P* = 0.032), tumor size (≥5 cm, HR = 1.395; *P* = 0.004), pLNR (>8%, HR = 8.192; *P* = 0.004), and liver metastasis (positive, HR = 1.368; *P* = 0.036; Supplementary Table 4).

### Novel Prognostic Nomogram for CSS Prediction

**Figure 4:**
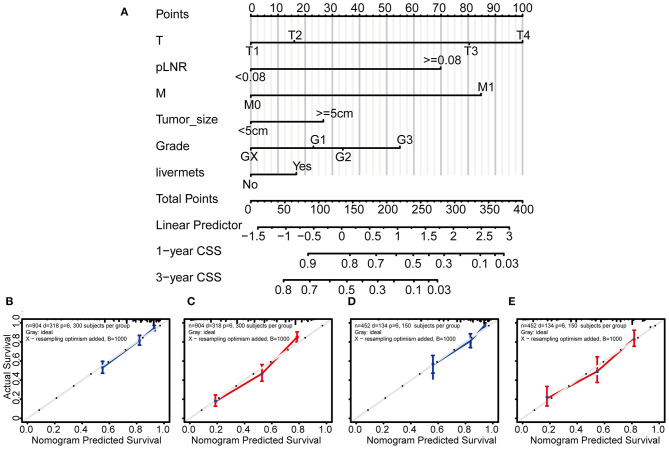
Nomogram and calibration curves for predicting the 1- and 3-year probabilities of cancer-specific survival in patients with gallbladder cancer. Grade: GX, Grade cannot be assessed; G1, well-differentiated; G2, moderately differentiated; G3, poorly differentiated. Nomogram **(A)**, calibration curves for predicting the 1- and 3-year probabilities of CSS in the training cohort **(B,C)** and in the validation cohort **(D,E)**. All the points identified on the top point scale for each factor were summed together to generate a total-point score. The total points projected on the bottom scales were used to determine the 1- and 3-year probabilities of CSS in individuals. The nomogram-predicted probability of survival is plotted on the X-axis, and the actual survival rate is plotted on the Y-axis. Vertical bars indicate 95% confidence intervals measured by Kaplan-Meier analysis.

Based on the above independent risk factors identified in the multivariate regression analysis and competing risk model analysis, we built a novel prognostic nomogram that combined T classification, pLNR, M classification, histologic grade, live metastasis, and tumor size (Figure 4A). Furthermore, point assignments and prognostic scores for each variable in the nomogram models were calculated in Supplementary Table 5. According to the predictive nomogram, T classification had the largest contribution and was followed by M classification, pLNR, histologic grade, tumor size, and liver metastasis. As shown in the calibration plots, the probability of 1- and 3-year CSS observed in the training and internal validation cohorts indicated the best consistency with the nomogram-predicted CSS (Figures 4B–E). The c-index for the CSS prediction nomogram was 0.763 in the training cohort, while the *c*-indexes for the AJCC 8th edition staging system, the AJCC 7th edition staging system, and the AJCC 6th edition staging system for CSS prediction were 0.718, 0.718, and 0.717, respectively, and were therefore much lower than those of the nomogram model. Similarly, in the validation cohort, the *c*-index was 0.783, which was higher than that of the AJCC staging system (Supplementary Table 6).

### Clinical Applications of the Formulated Nomogram Staging System

**Figure 5:**
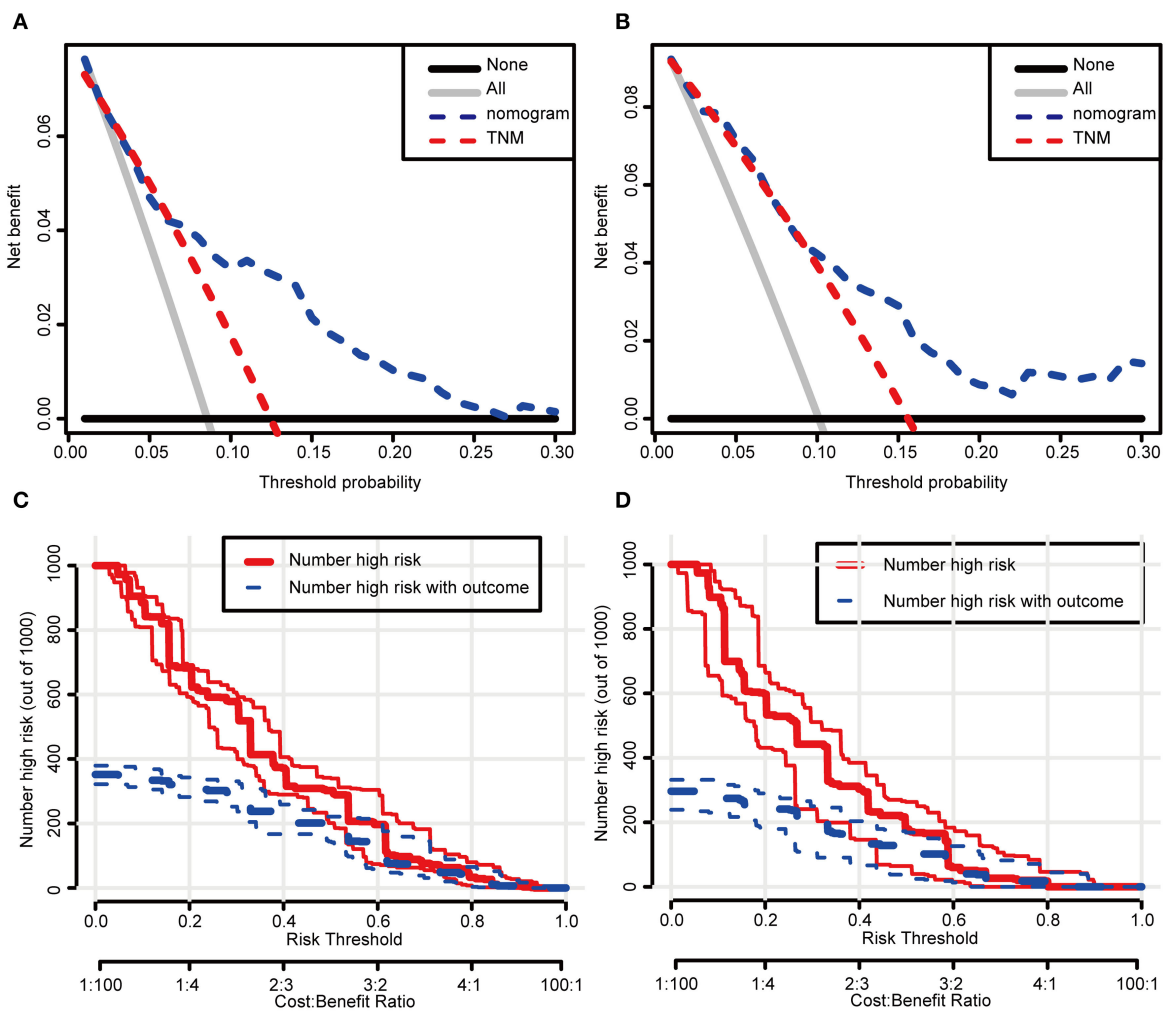
Decision curve and clinical impact curve analysis of the predictive nomogram. The nomograms were compared against TNM staging in terms of 3-year CSS in the training cohort **(A)** and the validation cohort **(B)** by DCA analysis. Clinical impact curves of the nomograms for CSS in the training cohort **(C)** and the validation cohort **(D)**. **(A,B)** The dashed lines indicate the net benefit of the models across a range of threshold probabilities. The horizontal solid black line represents the hypothesis that no patients experienced the endpoint, and the solid gray line represents the hypothesis that all patients met the endpoint. **(C,D)** At different threshold probabilities within a given population, the number of high-risk patients and the number of high-risk patients with the outcome were plotted.

**Figure 6:**
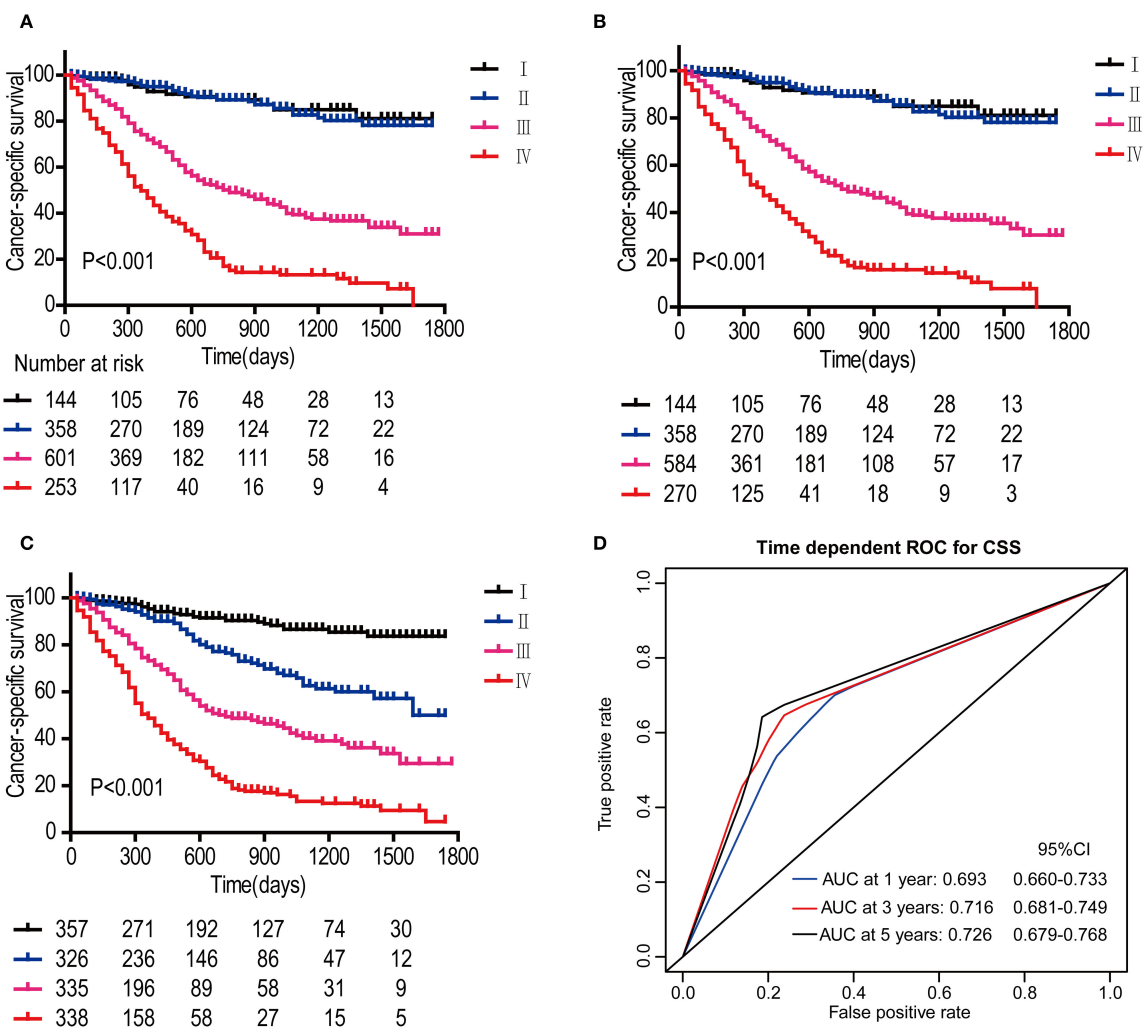
Kaplan-Meier survival curves for patients according to the AJCC 7th edition staging system **(A)**, AJCC 8th edition staging system **(B)**, and the formulated nomogram staging system **(C)** in the entire SEER cohort. *P*-values were determined by the log-rank test. Time-dependent ROC curve analysis of the formulated nomogram staging system in the entire SEER cohort **(D)**. The AUCs at 1, 3, and 5 years were calculated, and the 95% CIs were estimated with bootstrap methods. The *P*-values were two-sided; 95% CI, 95% confidence interval.

In the DCA, the results showed that the nomogram indicated a better net benefit than was achieved with the AJCC 8th edition staging system for predicting CSS in the training cohort (Figure 5A) and validation cohort (Figure 5B). Based on these DCAs, we further plotted curves to evaluate the clinical impact of the nomogram to help us more intuitively understand its significance value. Clinical impact curves of the nomogram to predict CSS in the training cohort (Figure 5C) and validation cohort (Figure 5D) showed great prediction abilities when the risk threshold was nearly <0.6. According to the formulated nomogram, all patients were divided into three groups according to optimal cutoff points determined by X-tile software (nomogram-stage I: <66, nomogram-stage II: 66–130, nomogram-stage III: 131–184, nomogram-stage IV: 185–354). There were 357, 326, 335, and 338 patients with nomogram stages I, II, III, and IV, respectively. Kaplan-Meier curves for CSS were plotted for the entire SEER cohort (Figure 6C) compared with the AJCC 7th edition staging system and the AJCC 8th edition staging system (Figures 6A,B). Based on the formulated nomogram staging, the discrimination between stage I and II disease was more appreciable than that of the AJCC staging system, in which the two curves were almost stuck together. The HRs of nomogram-stages II, III, and IV relative to nomogram-stage I in the entire SEER cohort based on univariate regression analysis were 2.796 (95% CI: 1.841–4.245, *P* < 0.001), 6.593 (95% CI: 4.474–9.713, *P* < 00.001), and 14.758 (95% CI: 10.151–21.456, *P* < 0.001), respectively. To compare the sensitivities and specificities of CSS the predictions, we used a time-dependent ROC curve analysis to assess AUCs at one, three, and 5 years for the formulated nomogram staging, which were 0.693, 0.716, and 0.726 in the entire SEER cohort, respectively (Figure 6D). All the above results suggested that the formulated nomogram staging was a stable predictor for CSS prediction with significant clinical value.

## Discussion

The present study shows that pLNR was an independent prognostic predictor for CSS in resected GBC patients based on Cox univariate and multivariate analyses. The majority of independent prognostic factors of CSS in the nomogram model identified by our study, including T classification, M classification, histologic grade, and tumor size, were all independent factors according to our work, which was consistent with a number of previous studies (11, 29–31).

For now, the prognostic indicators for GBC mainly include TNM staging (32), whether the surgical margin is positive, histological differentiation grade, related serological indicators such as CA199 (33), CEA (34) and so on. Although serological indicators are easy to obtain and can be monitored dynamically, they are less specific because they are also markers of other digestive system tumors and GBC-specific indicators have yet to be developed. Therefore, a pathological examination is important for evaluating the prognosis of patients undergoing GBC resection.

Lymph node metastasis is widely accepted to be associated with the prognosis of cancer patients. In the current TNM staging system, the parameter of the number of positive lymph nodes, is widely used to evaluate lymph node metastasis. The latest AJCC staging system recommends a minimum of 6 lymph nodes to accurately determine the N classification. Due to the characteristics of GBC, some of the patients diagnosed during surgery were originally considered to have a benign disease, and some patients were even diagnosed by pathological examination after surgery. For these patients, the number of resected lymph nodes may not meet the above criteria, the median and interquartile range of the number of total lymph nodes resected in group N0, N1, and N2 is 2 (1, 5), 2 (1, 5), 8 (6, 15), which means that most of the cases in N0 and N1 group can only be classified as Nx, and thus cause a stage migration phenomenon. The pLNR, which is widely used in a variety of malignancies (14–19), is less influenced by this variability and can act as an independent indicator to predict the prognosis of cancer patients. It seems reasonable to replace the number of positive lymph nodes with the pLNR to assess the lymph node status. When lymphadenectomy for GBC is standardized, i.e., we can harvest at least 6 lymph nodes from each procedure to send for examination, then it is more appropriate to compare the prognostic performance of pLNR with the number of positive lymph nodes through a large cohort.

The diagnosis of GBC is usually determined by intraoperative or postoperative pathological examination, and it is often in the moderate and advanced stages, requiring radical resection and lymphadenectomy, or even palliative treatment if resection is not possible (35). Cases with a high preoperative suspicion of gallbladder cancer should seek open surgery, as a good surgical field will guarantee maximal resection. However, the reality is that many patients usually undergo a cholecystectomy for what is initially diagnosed as a benign disease, which is often preferred as a laparoscopic cholecystectomy. The laparoscopic lymphadenectomy could not harvest a sufficient number of lymph nodes due to field limitations and technical limitations of the operator (36). At the same time, some scholars believe that laparoscopic cholecystectomy combined with partial hepatectomy and lymph node dissection can easily cause tumor metastasis in the abdominal cavity and metastasis of the Trocar sinus tract (37, 38). Ratti et al. showed that minimally invasive approach (MILS) radical GBC resection combined lymphadenectomy can achieve the same or better number of lymph nodes resected compared to the open approach without affecting lymphadenectomy-related complications and DFS, suggesting that laparoscopic resection of complex tumors and lymphadenectomy is practically feasible as a standardized procedure and applied in the clinic (39).

According to the present study, the number of positive lymph nodes does not show the expected value in guiding the prognosis of GBC patients. One possible explanation may be that although the number of positive lymph nodes is one of the risk factors, it is not as important as others; thus, its role is partially diluted in the multivariate analysis. Moreover, the pLNR showed stability in predicting patient CSS in the Cox regression analysis and competing risk model. Collectively, the use of the pLNR instead of the number of positive lymph nodes may be more convincing in assessing lymph node metastasis.

Interestingly, the advanced N classification (HR, 0.135; 95% CI, 0.03–0.615; *P* = 0.005) had an HR smaller than 1 in the Cox multivariate analysis, which is contrary to our usual findings, while the advanced N classification had an HR>1 (N1: HR = 3.115, *P* < 0.001; N2: HR = 3.978, *P* < 0.001) with reference to N0 in the Cox univariate analysis. The potential reason was that the roles of N classification would decrease considerably when combined with other important risk factors such as T classification in the SEER database. Surprisingly, pLNR>8% showed great harm for CSS (HR = 8.192; *P* = 0.004) in the Cox multivariate analysis, so we next built a novel nomogram staging based on the pLNR instead of the traditional N classification.

As a quantitative predictive model, the nomogram had a strong ability to predict survival and thus has the opportunity to replace the current TNM staging system (40–42). According to this study, LNR, T stage, M stage, histologic grade, tumor size, and liver metastasis are considered to be independent risk factors for GBC. The 5-year survival rates range from 70% with involvement of the subserosa (T2 = invasion of perimuscular connective tissue), decreasing to 0% with spread to adjacent organs (T3 = penetration of the serosa) (5, 43), and thus, T classification was the significant prognostic factor for GBC patients. Tumor differentiation represented the biological characteristics of GBC, and the median survival time of highly and, moderately differentiated GBC is significantly higher than that of poorly differentiated GBC (44). Adenocarcinoma is the most common histologic type, accounting for 98% of all GBC patients, and two-thirds of these tumors are moderately, or poorly differentiated (45). However, in this study, adenocarcinoma had an HR smaller than 1 in the Cox univariate analysis because a bias existed for the few other histologic types. Liver metastasis may contribute to this difference between the various anatomical locations (46). Thus, the formulated nomogram merged T, pLNR, M status and other significant factors together to construct a more precise model along with the validation consistent calibration curves and wider ranges of DCA.

Based on the formulated nomogram staging system, we found that the HRs for patients in each stage (HRs for stages II, III, and IV: 2.796, 6.593, and 14.758, respectively, with reference to stage I) were larger than those for the AJCC 8th edition staging system and the AJCC 7th edition staging system, as did the discrimination ability in the survival curves. The most important advantage of the formulated nomogram is the perfect discrimination between each stage compared with that of the AJCC 7th and 8th edition staging systems. This stratification is significant because only patients with stage II or higher disease can benefit from adjuvant therapy (9).

The present study has several merits. First, a large population from the international SEER database and not a dataset from a single institution was used to avoid heterogeneity among different medical centers. Second, the variables involved in the nomogram models are available and easy to obtain in routine clinical practice. Third, on the basis of the DCAs, we plotted curves for the clinical impact of the nomograms, and this helped us more intuitively understand its significance value in a clinical setting. A limitation of the study was its retrospective nature with selective bias, which requires a large-scale and multicenter prospective study. The type and extent of surgery can greatly affect the prognosis of patients with GBC. But the SEER database of GBC patients does not contain detailed information on the specific surgery procedures performed on the patient. Also, patients had a minimum survival time of 1 month, and the time between postoperative recovery and discharge from hospital (i.e., specific days of perioperative period) was not recorded, making it impossible to count non-tumor-related deaths during the perioperative period. So a prospective study based on the same inclusion and exclusion criteria is required to further confirm the reliability of the models, and additional Chinese centers are currently being recruited to build external validation datasets, at that time more details of the patients' surgical methods, surgical scope and degree, peri-operative complications, postoperative treatment and follow-up information, short-term and long-term prognosis and so on will be further studied and discussed. Additionally, the pLNR needs a meta-analysis to determine the most accurate cutoff value that is suitable for other studies. We hope to do so in a future investigation.

## Conclusions

Based on the clinical risk factors identified in a large population-based cohort, especially the pLNR, a robust prognostic predictor of CSS for resected GBC patients, we established the first practical formulated nomogram staging system that includes the T classification, pLNR, N classification, tumor size, histologic grade, and liver metastasis. Moreover, the internal validation cohort validation results demonstrated that these nomograms performed very well and showed excellent discrimination compared with the AJCC 7th and 8th edition staging systems. Our nomogram were demonstrated to be clinically useful in a DCA, and they should therefore help clinicians generate better risk stratifications and formulate individual treatments.

## Data Availability Statement

All datasets generated for this study are included in the article/Supplementary Material.

## Ethics Statement

The studies involving human participants were reviewed and approved by Medicine Institutional Review Board of Sun Yat-sen Memorial Hospital, Sun Yat-sen University. The ethics committee waived the requirement of written informed consent for participation.

## Author Contributions

KM and ZX: conception and design. KM, JW, and ZX: financial support. MZ, HL, and QZ: provision of study materials or patients. YY, JL, RC, KW, and JW: data analysis and interpretation. YY, JL, and MZ: manuscript writing. All authors: final approval of the manuscript.

## Conflict of Interest

The authors declare that the research was conducted in the absence of any commercial or financial relationships that could be construed as a potential conflict of interest.
