# Supplementary material for: A Novel Staging System to Forecast the Cancer-Specific Survival of Patients With Resected Gallbladder Cancer
Source: Front Oncol. 2020 Jul 28;10:1281. doi: 10.3389/fonc.2020.01281 (PMC7399135; doi:10.3389/fonc.2020.01281)
Supplement: Supplementary file 1 [file Data_Sheet_1.docx]

**Supplementary Table 1 Demographic and clinical characteristics of gallbladder carcinoma patients.**

| Clinicopathological Variables | | SEER Cohort (n=1356) | | | P Value |
| --- | --- | --- | --- | --- | --- |
|  |  | Entire cohort | Training (n=904) | Validation (n=452) |  |
| Age | <40 | 19 | 12 | 7 | 0.99 |
|  | 40-60 | 330 | 220 | 110 |  |
|  | 60-80 | 786 | 524 | 262 |  |
|  | ≥80 | 221 | 148 | 73 |  |
| Gender | Female | 942 | 632 | 310 | 0.661 |
|  | Male | 414 | 272 | 142 |  |
| Race | White | 1046 | 690 | 356 | 0.109 |
|  | Asian or Pacific Islander | 143 | 104 | 39 |  |
|  | Black | 155 | 105 | 50 |  |
|  | American Indian/Alaska Native | 12 | 5 | 7 |  |
| Marriage | Married | 728 | 490 | 238 | 1 |
|  | Single | 580 | 390 | 190 |  |
| Histologic type | Adenocarcinoma | 1049 | 697 | 352 | 0.783 |
|  | Other | 307 | 207 | 100 |  |
| Histologic grade | GX | 73 | 52 | 21 | 0.749 |
|  | G1 | 190 | 124 | 66 |  |
|  | G2 | 624 | 411 | 213 |  |
|  | G3 | 469 | 317 | 152 |  |
| AJCC 7^th^ edition stage | Ⅰ | 144 | 104 | 40 | 0.183 |
|  | Ⅱ | 358 | 226 | 132 |  |
|  | Ⅲ | 601 | 407 | 194 |  |
|  | Ⅳ | 253 | 167 | 86 |  |
| AJCC 8^th^ edition stage | Ⅰ | 144 | 104 | 40 | 0.177 |
|  | Ⅱ | 358 | 226 | 132 |  |
|  | Ⅲ | 584 | 391 | 193 |  |
|  | Ⅳ | 270 | 183 | 87 |  |
| T classification | T1+T2 | 834 | 558 | 276 | 0.859 |
|  | T3+T4 | 522 | 346 | 176 |  |
| N classification | N0 | 714 | 465 | 249 | 0.191 |
|  | N1 | 573 | 387 | 186 |  |
|  | N2 | 69 | 52 | 17 |  |
| M classification | M0 | 1166 | 781 | 385 | 0.599 |
|  | M1 | 190 | 123 | 67 |  |
| Tumor size | ﹤5 cm | 827 | 553 | 274 | 0.890 |
|  | ≥5 cm | 529 | 351 | 178 |  |
| Tumor extension | 1 | 165 | 119 | 46 | 0.381 |
|  | 2 | 606 | 399 | 207 |  |
|  | 3 | 433 | 282 | 151 |  |
|  | 4 | 152 | 104 | 48 |  |
| pLNR | ≤8% | 731 | 473 | 258 | 0.109 |
|  | ﹥8% | 625 | 431 | 194 |  |
| Liver metastasis | Negative | 1221 | 817 | 404 | 0.565 |
|  | Positive | 135 | 87 | 48 |  |
| Status | Alive | 618 | 394 | 224 | 0.109 |
|  | CSS | 686 | 473 | 213 |  |
|  | Other death | 52 | 37 | 15 |  |

pLNR, positive lymph node ratio; tumor extension, 1: tumor invades the lamina propria or muscular layer; 2: tumor invades the perimuscular connective tissue; 3: tumor invades the liver; 4: tumor invades extrahepatic organs or structures.

**Supplementary Table 2 Correlations between pLNR and clinical characteristics of patients with**

**resected gallbladder carcinoma in the SEER cohort.**

| Clinical  characteristics | | SEER cohort | | P value |
| --- | --- | --- | --- | --- |
|  |  | pLNR≤0.08(n=731) | pLNR>0.08(n=625) |  |
| Age |  | 67.38(12.30) | 66.91(11.49) | 0.068 |
| Gender | Male | 231 | 183 | 0.386 |
|  | Female | 500 | 442 |  |
| Marriage | Married | 376 | 352 | 0.113 |
|  | Single | 326 | 254 |  |
| Race | Asian or Pacific Islander | 76 | 67 | 0.556 |
|  | American Indian/Alaska Native | 8 | 4 |  |
|  | White | 557 | 489 |  |
|  | Black | 90 | 65 |  |
| Histologic type | Adenocarcinoma | 588 | 461 | **0.004** |
|  | Other | 143 | 164 |  |
| Histologic grade | GX | 46 | 27 | **<0.001** |
|  | G1 | 142 | 48 |  |
|  | G2 | 359 | 265 |  |
|  | G3 | 184 | 285 |  |
| AJCC 7^th^ edition stage | I | 144 | 0 | **<0.001** |
|  | II | 358 | 0 |  |
|  | III | 158 | 443 |  |
|  | IV | 71 | 182 |  |
| AJCC 8^th^ edition stage | I | 149 | 16 | **<0.001** |
|  | II | 388 | 281 |  |
|  | III | 183 | 299 |  |
|  | IV | 11 | 29 |  |
| N classification | N0 | 714 | 0 | **<0.001** |
|  | N1 | 17 | 556 |  |
|  | N2 | 0 | 69 |  |
| M classification | M0 | 667 | 499 | **<0.001** |
|  | M1 | 64 | 126 |  |
| Tumor size | ﹤5 cm | 471 | 356 | **0.006** |
|  | ≥5 cm | 260 | 269 |  |
| Tumor extension | 1 | 149 | 16 | **<0.001** |
|  | 2 | 353 | 253 |  |
|  | 3 | 184 | 249 |  |
|  | 4 | 45 | 107 |  |
| Bone metastasis | Negative | 731 | 618 | **0.004** |
|  | Positive | 0 | 5 |  |
|  | Unknown | 0 | 2 |  |
| Brain metastasis | Negative | 731 | 620 | **0.021** |
|  | Positive | 0 | 1 |  |
|  | Unknown | 0 | 4 |  |
| Liver metastasis | Negative | 686 | 535 | **<0.001** |
|  | Positive | 45 | 90 |  |
| Lung metastasis | Negative | 731 | 613 | **<0.001** |
|  | Positive | 0 | 10 |  |
|  | Unknown | 0 | 2 |  |

Bold values: Statistical differences are significant. tumor extension, 1: tumor invades the lamina propria or muscular layer; 2: tumor invades the perimuscular connective tissue; 3: tumor invades the liver; 4: tumor invades extrahepatic organs or structures.

**Supplementary Table 3 Univariate regression analysis of risk factors associated with cancer specific survival of patients with resected gallbladder carcinoma in the training cohort.**

| **Variables** | HR | 95% CI | *P* value | **Variables** | HR | 95% CI | *P* value |
| --- | --- | --- | --- | --- | --- | --- | --- |
| Age |  |  |  | Histologic grade |  |  |  |
| <40 | 1 |  |  | GX | 1 |  |  |
| 40-60 | 0.727 | 0.317-1.665 | 0.45 | G1 | 0.838 | 0.436-1.612 | 0.596 |
| 60-80 | 0.737 | 0.320-1.664 | 0.463 | G2 | 1.352 | 0.780-2.345 | 0.283 |
| ≥80 | 0.73 | 0.312-1.712 | 0.47 | G3 | 2.379 | 1.374-4.118 | **0.002** |
| Gender |  |  |  | AJCC 7^th^ edition stage |  |  |  |
| Female | 1 |  |  | Ⅰ | 1 |  |  |
| Male | 0.996 | 0.782-1.268 | 0.974 | Ⅱ | 1.146 | 0.569-2.711 | 0.702 |
| Marriage |  |  |  | Ⅲ | 5.168 | 2.807-9.517 | **<0.001** |
| Married | 1 |  |  | Ⅳ | 13.109 | 7.03-24.445 | **<0.001** |
| Single | 0.995 | 0.762-1.196 | 0.686 | AJCC 8^th^ edition stage |  |  |  |
| Race |  |  |  | Ⅰ | 1 |  |  |
| Asian or Pacific Islander | 1 |  |  | Ⅱ | 1.146 | 0.568-2.311 | 0.703 |
| American Indian/Alaska Native | 0.867 | 0.210-3.584 | 0.844 | Ⅲ | 5.09 | 2.762-9.382 | **<0.001** |
| White | 0.771 | 0.556-1.071 | 0.121 | Ⅳ | 12.388 | 6.659-23.046 | **<0.001** |
| Black | 0.755 | 0.482-1.183 | 0.22 | T classification |  |  |  |
| Tumor size |  |  |  | T1-2 | 1 |  |  |
| ﹤5 cm | 1 |  |  | T3-4 | 3.595 | 2.86-4.519 | **<0.001** |
| ≥5 cm | 1.67 | 1.34-2.082 | **<0.001** | N classification |  |  |  |
| Tumor expansion |  |  |  | N0 | 1 |  |  |
| 1 | 1 |  |  | N1 | 3.115 | 2.434-3.987 | **<0.001** |
| 2 | 1.845 | 1.101-3.091 | **0.02** | N2 | 3.978 | 2.659-5.951 | **<0.001** |
| 3 | 4.555 | 2.745-7.556 | **<0.001** | M classification |  |  |  |
| 4 | 7.728 | 4.553-13.118 | **<0.001** | M0 | 1 |  |  |
| pLNR |  |  |  | M1 | 4.309 | 3.332-5.574 | **<0.001** |
| ≤8% | 1 |  |  | Histologic type |  |  |  |
| >8% | 3.314 | 2.608-4.213 | **<0.001** | Adenocarcinoma | 1 |  |  |
| Liver metastasis |  |  |  | Other | 1.401 | 1.097-1.789 | **0.007** |
| Negative | 1 |  |  |  |  |  |  |
| Positive | 4.407 | 3.296-5.894 | **<0.001** |  |  |  |  |

Bold values: Statistical differences are significant. pLNR, positive lymph node ratio; tumor extension, 1: tumor invades lamina propria or muscular layer; 2: tumor invades the perimuscular connective tissue; 3: tumor invades the liver; 4: tumor invades extrahepatic organs or structures.HR: hazard ratio; 95% CI, 95% confidence interval.

**Supplementary Table 4 Multivariate regression analysis of risk factors associated with cancer-specific survival of patients with resected gallbladder carcinoma in the training cohort.**

| Variables | HR | 95% CI | | P value |
| --- | --- | --- | --- | --- |
|  |  | Lower | Upper |  |
| Histologic grade |  |  |  | **0.025** |
| GX | 1 |  |  |  |
| G1 | 1.33 | 0.687 | 2.576 | 0.398 |
| G2 | 1.566 | 0.895 | 2.74 | 0.116 |
| G3 | 1.958 | 1.123 | 3.415 | **0.018** |
| AJCC 8^th^ edition stage |  |  |  | **0.001** |
| Ⅰ | 1 |  |  |  |
| Ⅱ | 1.059 | 0.524 | 2.141 | 0.873 |
| Ⅲ | 1.944 | 0.968 | 3.904 | 0.062 |
| Ⅳ | 4.028 | 1.753 | 9.257 | **0.001** |
| T classification |  |  |  |  |
| T1+2 | 1 |  |  |  |
| T3-4 | 1.888 | 1.446 | 2.463 | **<0.001** |
| N classification |  |  |  | **0.005** |
| N0 | 1 |  |  |  |
| N1 | 0.211 | 0.051 | 0.874 | **0.032** |
| N2 | 0.135 | 0.03 | 0.615 | **0.01** |
| M classification |  |  |  |  |
| M0 | 1 |  |  |  |
| M1 | 1.696 | 1.047 | 2.748 | **0.032** |
| Tumor size |  |  |  |  |
| ﹤5 cm | 1 |  |  |  |
| ≥5 cm | 1.395 | 1.114 | 1.748 | **0.004** |
| pLNR |  |  |  |  |
| ≤8% | 1 |  |  |  |
| >8% | 8.192 | 1.994 | 33.665 | **0.004** |
| Liver metastasis |  |  |  |  |
| Negative | 1 |  |  |  |
| Positive | 1.368 | 1.078 | 2.325 | **0.036** |

Bold values: Statistical differences are significant. pLNR, positive lymph node ratio; HR: hazard ratio; 95% CI, 95% confidence interval.

**Supplementary Table 5 Point assignments and prognostic scores for each variable in the nomogram model**

| Variables | Classification | Nomogram score |
| --- | --- | --- |
| Histologic grade | GX | 0 |
|  | G1 | 23 |
|  | G2 | 34 |
|  | G3 | 55 |
| T classification | T1 | 0 |
|  | T2 | 16 |
|  | T3 | 80 |
|  | T4 | 100 |
| pLNR | ≤8% | 0 |
|  | >8% | 70 |
| M classification | M0 | 0 |
|  | M1 | 85 |
| Liver metastasis | Negative | 0 |
|  | Positive | 17 |
| Tumor size | ﹤5 cm | 0 |
|  | ≥5 cm | 27 |

pLNR, positive lymph node ratio

**Supplementary Table 6 The c-index for the nomogram to predict CSS**

| Variable | Training set (n=904) | | Testing set (n=452) | |
| --- | --- | --- | --- | --- |
|  | c-index | 95% CI | c-index | 95% CI |
| Nomogram stage | 0.763 | 0.728-0.798 | 0.783 | 0.728-0.838 |
| T+pLNR+M | 0.746 | 0.711-0.781 | 0.759 | 0.706-0.812 |
| AJCC 8^th^ edition stage | 0.718 | 0.685-0.751 | 0.745 | 0.694-0.796 |
| AJCC 7^th^ edition stage | 0.718 | 0.685-0.751 | 0.743 | 0.692-0.794 |
| AJCC 6^th^ edition stage | 0.717 | 0.686-0.748 | 0.743 | 0.692-0.794 |
| Histologic grade | 0.6 | 0.567-0.633 | 0.666 | 0.615-0.717 |
| pLNR | 0.646 | 0.615-0.677 | 0.67 | 0.623-0.717 |
| Tumor size | 0.57 | 0.541-0.599 | 0.569 | 0.522-0.616 |

pLNR, positive lymph node ratio
